# "Raising a Ghost"—And Laying a Controversy

**Published:** 1917-11-03

**Authors:** 


### The Editor's Letter-Box.

### "Raising a Ghost"—and Laying a Controversy.

*To the Editor of* THE HOSPITAL.

SIR,—Dr. Hawthorne's lengthy reply amounts to this: That the B.M.A. has claimed payment for professional attendance upon discharged, disabled men (from the Local Pensions Committees), and not for attendance upon the wounded (from the War Office), because some V.A.D. practitioners have demanded it in the case of the former, while apparently their colleagues on the hospital staffs in respect of the latter have not. But this, perspicuous and probable so far as it goes, does not explain the motive which moved the V.A.D. practitioners to their demand, nor why it did not also prompt their colleagues on the staffs. The position of the profession towards both classes of men is the same in principle. It is only the authority which controls the proposed payment which is different. Therefore, in default of a better, our explanation of the motive which prompted the action of one section of the profession, but which has not prompted similar action under similar circumstances in another section, still holds the field. It is because we do not "again refer" to our "literary gems," nor to our correspondents' periphrases which we could not honestly describe as gems, that we can be the briefer.—Yours faithfully,

THE WRITER OF THE ARTICLE.

